# Allo-HSCT recipients with invasive fungal disease and ongoing immunosuppression have a high risk for developing tuberculosis

**DOI:** 10.1038/s41598-019-56013-w

**Published:** 2019-12-31

**Authors:** Apeng Yang, Jimin Shi, Yi Luo, Yishan Ye, Yamin Tan, He Huang, Yanmin Zhao

**Affiliations:** 1 Bone Marrow Transplantation Center, The First Affiliated Hospital, School of Medicine, Zhejiang University, Hangzhou, 310003 China; 2 Institute of Hematology, Zhejiang University, Hangzhou, 310003 China; 3 Department of Hematology, The First Affiliated Hospital of Fujian Medical University, Fuzhou, 350005 China

**Keywords:** Risk factors, Signs and symptoms

## Abstract

Patients underwent allogeneic hematopoietic stem cell transplantation (allo-HSCT) are at high risk of acquiring tuberculosis (TB) due to a status of immunosuppression. We conducted a nested case control study to investigate the incidence and risk factors for TB after allo-HSCT. Between 2012 and 2017, 730 consecutive allo-HSCT recipients were enrolled, and 14 patients (1.92%) were diagnosed with TB. Relatively, 54 allo-HSCT recipients were selected as control. Patients who suffered TB had a significantly higher 3-year non-relapse mortality rate than the control group (30.36% vs 5.39%, P < 0.01). In multivariate analysis, invasive fungal disease (HR 4.87, 95% CI 1.39–17.09), treatment with a relatively high dose of prednisone (HR 10.34, 95% CI 1.12–95.47) and treatment with tacrolimus (HR 4.79, 95% CI 1.18–19.44) were identified independent risk factors for TB occurrence post allo-HSCT (P < 0.05). Meanwhile, donor type, dose and type of anti-thymocyte globulin (ATG) administrated, as well as treatment intensity, did not alter the incidence of TB. Therefore, allo-HSCT recipients with unexplained fever, especially those who suffer from invasive fungal disease and ongoing immunosuppression with a relatively high dose of prednisone or tacrolimus, are at a high-risk of developing active TB. Closely Monitoring TB occurrence, making a timely diagnosis and administering the proper treatment may be beneficial to those high-risk patients.

## Introduction

Tuberculosis (TB) remains one of the biggest threats for global public health. More than 2 billion people worldwide are infected with *Mycobacterium tuberculosis* (*M. tuberculosis*) and it was responsible for nearly 1.3 million deaths in 2012^1–3^. China currently has approximately 1,000,000 TB cases, ranking second among all countries in the world. Moreover, patients with immunosuppression are at a higher risk of acquiring TB, leading to significant morbidity and mortality^4^.

For recipients of hematopoietic stem cell transplantation (HSCT), cellular immunity is extremely disrupted by high-dose chemoradiotherapy and subsequent immunosuppressive therapies. Immune reconstitution is delayed after engraftment and will not be completed until 1 to 2 years post-HSCT. Moreover, in the condition of allogeneic HSCT (allo-HSCT), Graft-versus-host disease (GVHD) develops frequently, leading to further deterioration of cellular immunity due to dysregulation and abnormal clonal expansion of T cells^5^. Opportunistic infections caused by bacteria, viruses, and fungi can occur at this time^6^.

Previous observational studies have reported an incidence of 0.80% to 2.84% for TB post-HSCT in the most recent decade^7–10^. So far, the disease features of TB after allo-HSCT have not been thoroughly described, and the risk factors leading to the occurrence of TB have not been identified. Early diagnosis should be emphasized, and timely treatments should be developed for post-HSCT tuberculosis in order to improve theclinical outcomes. Thus, we conducted a nested case control study to identify the disease features and risk factors for TB after allo-HSCT.

## Materials and Methods

### Patients

All patients who received allo-HSCT from January 2012 to December 2017 for hematologic diseases were examined. The diagnosis of TB was based on published criteria^8^. Clinical data were collected after the peripheral blood stem cell (PBSC) reinfusion. The protocol was approved by the ethics review committee of the First Affiliated Hospital of Zhejiang University School of Medicine. All participants gave their written informed consent in accordance with the Declaration of Helsinki. Each patient had ongoing follow-up care until either October 31, 2018 or the last visit.

### Definition of *M. tuberculosis* infection

To confirm the diagnosis of active TB, sputum or tissue obtained must be smear-positive or culture positive, and acid-fast organisms and polymerace chain reaction (PCR) results must be positive for Mycobacterium tuberculosis. Miliary TB was diagnosed according to the criteria previous reported^11^. The radiological findings were confirmed by two experienced radiologists. Patients who matched above criteria were considered proven for M. tuberculosis infection. A patient was considered as a possible case when all the following criteria were met: 1. No definite evidence of other infectious. 2. The patient’s condition recovered with anti-TB treatment when other antibacterial and antifungal agents were ineffective^12–14^. 3. Interferon-gamma release assays (IGRA) (such as T-SPOT.TB) positive or a switch from negative to positive^15–17^. The date of TB diagnosis was defined when all the criteria were met.

A latent tuberculosis infection (LTBI) was defined as a state of persistent immune response to stimulation by *Mycobacterium tuberculosis* antigens with no evidence of clinically manifest active TB. Either IGRA or tuberculin skin test (TST) could be applicated to detect LTBI. There weren’t any signs or symptoms of TB in the vast majority of infected people but they were at risk of suffering active TB^18^.

### Definition of a relatively high dose of prednisone

The relatively high dose of prednisone was defined as: (1) A dose that started at 1–2 mg/kg/day without dose tapering for at least two weeks; or (2) A dose that started at 1–2 mg/kg/day and then tapered to no less than 0.5 mg/kg/day for more than 6 weeks.

### GVHD

All patients received GVHD prophylaxis consisting of cyclosporin A (CSA) and short-term methotrexate (MTX) either with or without a low-dose mycophenolate mofetil (MMF). Tacrolimus (FK 506) was not routinely used in our department, while it may be added as a precautionary measure in case of CSA intolerance^19^. Diagnosis and grading of acute GVHD (aGVHD) was based on the consensus criteria^20^, and the definition of chronic GVHD was based on the revised Seattle classification^21^.

### Control selection

For each patient diagnosed with TB, 4 allo-HSCT recipients of the same gender and aged ±5 years were selected as control.

### Study end points

We compared the patients who developed TB with the control to investigate the risk factors for TB after allo-HSCT. The study end points were 3-year overall survival (OS), non-relapse mortality and TB-related mortality.

### Statistical analysis

Since the median time between transplant and TB occurrence was 193.5 days in our cohort, we chose all the clinical and laboratory signs, as well as transplantation complications, that presented within 193 days in the control group or before TB occurrence in the TB group as potential risk factors.

Medians and ranges were used to present continuous variables. For univariate analysis, Pearson’s Chi-square test, Chi-square test with continuity correction, and Fisher’s exacts were used to compare categorical variables under different circumstances. Variables with p ≤ 0.1 in the univariate analysis were introduced as risk factors and candidates for multivariate analysis. For multivariate analysis, we used conditional logistic regression with a backward likelihood ratio method, and variables were retained if p ≤ 0.05. The 3-year OS was generated using the Kaplan-Meier method. The 3-year NRM and TB-related mortality cumulative incidence curve was computed using Gray’s competing risk method, with relapse and death without TB as competing failure mechanisms. R statistical software (version 3.5.0) and SPSS (Version 22.0) were used for statistical analysis.

## Results

### Patient characteristics

The patient characteristics and risk factors for TB are shown in Table 1. Between January 2012 and December 2017, a total of 730 patients with acute lymphoblastic leukemia (ALL), acute myeloid leukemia (AML), chronic myeloid leukemia (CML), myelodysplastic syndrome (MDS), aggressive lymphoma or other hematologic diseases underwent allo-HSCT. The median follow-up duration of the survivors was 23.59 (range 3.89–88.21) months. During the studying period, 14 patients (1.92%) were diagnosed with active TB and 56 allo-HSCT recipients were selected as control. No significant difference was observed between the two groups concerning age, gender, underlying disease, donor type, with/without ATG administration or the conditioning intensity.

**Table 1:** Demographic characteristics of the 70 Allo-HSCT recipients and risk factors for TB occurrence.

70 Allo-HSCT recipients	Subjects with TB (n = 14)		Subjects without TB (n = 56)		P value
Demographic characteristics	27.5	9–60	29	9–65	
Age					0.988
Sex					0.923
Male	9	64.29%	36	64.29%	
Female	5	35.71%	20	35.71%	
Underlying disease					0.983
Acute lymphoblastic leukaemia	3	21.43%	24	42.86%	
Acute myeloblastic leukaemia	8	57.14%	20	35.71%	
hybrid acute leukemia	1	7.14%	1	1.79%	
Myelodysplastic syndrome	0	0.00%	6	10.71%	
Chronic myeloid leukaemia	1	7.14%	2	3.57%	
Aggressive lymphoma	1	7.14%	2	3.57%	
myelofibrosis	0	0.00%	1	1.79%	
Donor type					0.437
Unrelated donor	3	21.43%	9	16.07%	
Haploidentical-related donor	8	57.14%	31	55.36%	
Matched sibling donors	3	21.43%	16	28.57%	
ATG given as conditioning	12	85.71%	41	73.21%	0.305
Conditioning intensity					0.774
MAC	13	92.86%	52	92.86%	
RIC	1	7.14%	4	7.14%	
GVHD prophylaxis					
CSA based	5	35.71%	42	75.00%	0.344
FK 506 based	9	64.29%	14	25.00%	0.009
TB occurrence (months after HSCT)	6.95	1.54–32.46	N/A		
3-year overall survival after HSCT	8	57.14%	43	76.79%	0.102
NRM at 3 years after HSCT	5	30.36 ± 13.55%	3	5.39 ± 3.06%	0.002
aGVHD (%)					0.013
Grades0–1	5	35.71%	41	73.21%	
Grades2–4	9	64.29%	15	26.79%	
*cGVHD (%) group after HSCT ≥100d					0.033
none-mild	4	40%	32	80%	
moderate-severe	6	60%	8	20%	
Cytomegalovirus viremia	11	78.57%	30	53.57%	0.123
Epstein Barr virus viremia	13	92.86%	30	53.57%	0.032
Invasive fungal disease	11	78.57%	13	23.21%	0.012
Follow-up duration, median months (range)	18.27	3.93–88.21	27.14	3.89–87.93	0.219
Patients who died(range)	12.05	3.93–39.79	13.93	3.89–51.86	
Patients alive at last follow-up(range)	42.02	13.43–88.21	43.07	11.04–87.93	

MAC: myeloablative conditioning.

RIC: reduced intensity conditioning.

ATG: anti-thymocyte globulin.

CSA: Cyclosporine A.

FK 506: tacrolimus.

### Clinical characteristics of TB patients

**Figure 1:**
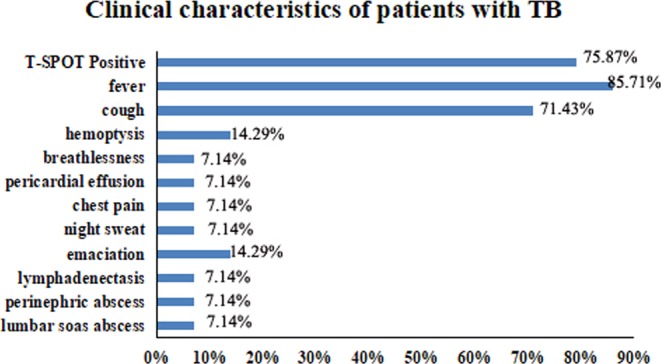
Clinical characteristics of patients with TB in our treatment facility.

All the evaluated subjects underwent CT screening before transplantation to exclude pretransplant tuberculosis. Having a positive T-SPOT or a transfer from negative to positive was indicative of the possible presence of tuberculosis. To distinguish de novo and recurrent TB after allo-HSCT, we excluded all the patients who were shown positive for the T-SPOT assay before the transplantation. Of the 14 patients with TB after allo-HSCT, 10 were confirmed and 4 were possible cases. The median interval from allo-HSCT to TB diagnosis was 193.5 (43 to 909) days. 12 of the 14 cases were pulmonary TB (85.71%), while the other 2 were extrapulmonary TB (14.29%). 4 patients suffered miliary TB. Unexplained fever (85.71%) and cough (71.43%) uncontrollable by conventional antibiotics were common in these patients. The T-SPOT positive rate was significantly higher in patients with TB than whose without (HR = 6.29, 95% CI, 3.09–12.77; P = 0.000) (Figure. 1).

13 patients (92.86%) received first-line anti-TB treatment (INH, rifampicin, ethambutol, and pyrazinamide) and 1 patient received second-line therapy because of INH and rifampicin resistance. 5 cases were susceptible to anti-TB medication, 4 cases were still under treatment at the end of the study period, and 5 cases did not respond to anti-TB treatment. 6 patients died within the study period, with 4 with respiratory failure, 1 with engraftment failure/multiorgan failure, and 1 with primary disease relapse while receiving anti-TB treatment. The clinical characteristics and diagnosis/treatment of the TB patients are summarized in Table S1.

### Risk factors for TB occurrence

In univariate analysis, we identified grades 2 to 4 aGVHD (HR = 3.98, 95% CI, 1.33–11.88; P = 0.013), moderate to severe cGVHD (HR = 3.95, 95% CI, 1.11–14.03; P = 0.033), Epstein Barr virus viremia (HR = 9.21; 95% CI, 1.20–70.45; P = 0.032), invasive fungal disease (HR = 3.91, 95% CI, 1.35–11.32; P = 0.012), treatment with etanercept (HR = 3.93, 95% CI, 1.38–11.22; P = 0.011), a relatively high dose of prednisone (HR = 17.83, 95% CI, 2.33–136.48, P = 0.006), and tacrolimus (HR = 4.34, 95% CI, 1.45–12.97, P = 0.009) as risk factors for the occurrence of TB. It is necessary to note that a possible diagnosis of cGVHD was investigated in patients diagnosed with TB 100 days post-transplantation and in controls who survived over 100 days after allo-HSCT. Therefore, 10 TB patients and 40 controls were studied for cGVHD.

**Figure 2:**
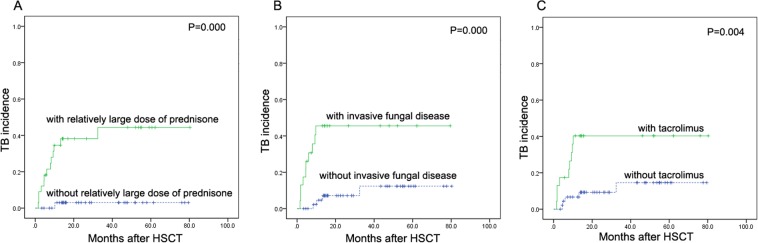
Independent risk factors of TB incidence (**A**). Curve comparing groups with or without treatment with a relatively high dose of prednisone (P = 0.000); (**B**) Curve comparing groups with or without invasive fungal disease (P = 0.000); (**C**) Curve comparing groups with or without treatment with tacrolimus (P = 0.004).

Multivariate analysis (Table 2) revealed that invasive fungal disease (HR 4.87, 95% CI 1.39–17.09), treatment with a relatively high dose of prednisone (HR 10.34, 95% CI 1.12–95.47) and tacrolimus (HR 4.79, 95% CI 1.18–19.44) were independent risk factors for TB occurrence (P < 0.05). Figure 2A–C shows the correlation of these risk factors with TB incidence after HSCT.

**Table 2:** Risk factors for tuberculosis in patients after allogeneic hematopoietic stem cell transplantation.

Predictive variables	Univariate analysis		Multi-variate analysis	
	HR (95%CI)	P value	HR (95%CI)	P value
Donor type	1.37 (0.62–3.04)	0.437		
Age, years (per year)	1 (0.96–1.04)	0.988		
Male sex	1.06 (0.35–3.15)			
Donor type	1.37 (0.62–3.04)	0.437		
aGVHD(grade ≥2)	3.98 (1.33–11.88)	0.013	1.12 (0.24–5.19)	0.881
cGVHD(moderate-severe)	3.95 (1.11–14.03)	0.033	0.31 (0.06–1.69)	0.175
GVHD prophylaxis				
CSA based	0.69 (0.32–1.48)	0.344		
FK 506 based	4.34 (1.45–12.97)	0.009	4.79 (1.18–19.44)	0.028
etanercept	3.93 (1.38–11.22)	0.011	0.81 (0.16–4.01)	0.797
relatively high dose of prednisone	17.83 (2.33–136.48)	0.006	10.34 (1.12–95.47)	0.039
Epstein Barr virus viremia	9.21 (1.20–70.45)	0.032	5.38 (0.66–43.61)	0.115
Invasive fungal disease	3.91 (1.35–11.32)	0.012	4.87 (1.39–17.09)	0.014

CSA: Cyclosporine A; FK 506: tacrolimus.

### Disease outcome

**Figure 3:**
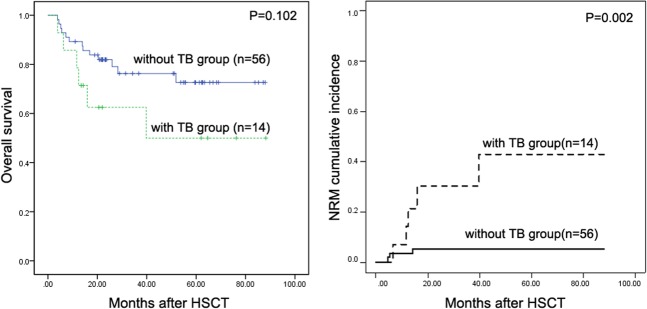
(**A**) Three-year overall survival (OS) rates comparing subjects with and without TB. No statistically significant difference was found between subjects with and without TB (57.14% vs 76.79%; P = 0.102); (**B**)Three-year nonrelapse mortality (NRM) cumulative incidence curve. Grey’s competing risk method revealed a significantly higher 3-year NRM in subjects with TB compared to that of subjects without TB (30.36% ± 13.55% vs 5.39% ± 3.06%, P = 0.002).

The median survival of patients with TB was 18.27 (range 3.93–88.21) months post-HSCT. The causes of death were TB/engraftment failure and multiorgan failure (n = 1), TB/primary disease relapse (n = 1), and TB/death due to respiratory failure (n = 4). Figure 3A shows the comparative 3-year OS rates. With the median follow-up of 18.27 (range 3.93–88.21) months for TB patients and 27.14 (range 3.89–87.93) months for patients without TB, the 3-year OS rates were 57.14% and 76.79%, respectively (P = 0.102). Figure 3B shows the 3-year NRM cumulative incidence curve, which shows that the 3-year NRM was significantly higher in TB patients than patients without TB (30.36% ± 13.55% vs 5.39% ± 3.06%, P = 0.002).

## Discussion

Post-transplantation TB infections are rare, but they carry significant morbidity and mortality. TB screening for recipients before allo-HSCT should be emphasized because active TB can either occur via reactivation of latent disease within the immunosuppressed recipient or via acquisition of new infections^22^. However, TB screening of donors is not recommended by the American Society of Blood and Marrow Transplantation (ASBMT) as donor-derived TB has not been reported in the HSCT setting^23^. In solid organ transplantation, however, donors with a TB infection should be excluded, as active tuberculosis could be transmitted from the donor’s infected organs (e.g., lung or liver) to the recipient^24–26^.

China has the second largest population of TB patients worldwide. The incidence of TB among allo-HSCT recipients is reported to be 2–40 times higher than that of the general population^9,12,27–29^. In our department, active TB occurred in 1.92% of patients underwent allo-HSCT, which is lower than that reported in other parts of Asia such as India, Pakistan and Taiwan, regardless of the fact that study periods and cohorts varied^7–10^. The lower incidence might be related to the single-agent INH prophylaxis for those recipients with positive T-SPOT before allo-HSCT. However, the true incidence of TB in the present study might be underestimated, because the TB diagnosis may be difficult given the nonspecific presentations of the disease, and a useful diagnostic approach-molecular testing for mycobacterial DNA (such as GeneXpert MTB/RIF) hasn’t been performed in our center.

In our study, the 3-year NRM was 4.35 times higher in TB patients than that in subjects without, indicating that active TB had a pivotal impact on their clinical outcomes. Therefore, TB risk factors should be screened and TB monitoring should be done soon after HSCT. Several risk factors for post-HSCT TB occurrence have been reported, including the use of busulfan, cyclophosphamide, corticosteroid therapy, tacrolimus, GVHD, etc. Herein, we revealed the significant predictive value of Epstein Barr virus viremia, invasive fungal disease, aGVHD (Grades 2 to 4), cGVHD (moderate-severe), treatment with etanercept, treatment with a relatively high dose of prednisone and tacrolimus for TB post allo-HSCT. Among them, aGVHD and cGVHD have been reported to be closely related to posttransplant TB in many studies^9,29^. However, The immune reactivity to EBV among patients with active TB has not been explored. Intact cellular immune responses to EBV reflect the general immunological fitness^30^. Compromised immune responses to EBV are linked to disease progression in patients with cancer or in patients posttransplantation^30^. Progression of latent TB infection to clinical TB disease is associated with aberrant host immune responses^31^. EBV antigens reported may represent as one of intrinsic markers for immune fitness during TB treatment^32^. Thus, EBV viremia might be a co-infection and co-morbidity that could be associated as one of TB risk factors. Herein, notably, we identified only three independent risk factors for the development of post-HSCT TB by a multivariate analysis: 1) the presence of high-risk invasive fungal disease (IFD); 2) treatment with a relatively high dose of prednisone; and 3) treatment with tacrolimus. It is known that the immunosuppressive activity of FK506 is 10 to 100 times more potent than CSA^33^. The inhibition of T lymphocyte activation and additional immunosuppression are frequently seen in cases where there is a long-term use of tacrolimus and a high dose of glucocorticoid^34,35^. Comorbid opportunistic infections such as IFD may act as potential risk for acquiring TB^36^.

Early diagnose of TB is very important for management and control but become a challenge in seriously immunocompromised hosts, such as HSCT recipients, in whom TB may present different clinical manifestation. A definite diagnosis of TB in patients who have received HSCT is usually difficult to establish because immunological deficits may lead to nonspecific clinical features. In addition to the above risk factors, low fever or chronic cough that is uncontrollable with common antibiotics and a positive T-SPOT (Fig. 1) were the specific and common manifestations of tuberculosis. HSCT recipients with these signs should be closely monitored for TB.

In conclusion, we propose that invasive fungal disease, treatment with a relatively high dose of prednisone, and tacrolimus were independent risk factors for the development of TB after HSCT. Strict standardized diagnostic standards and monitoring strategies, as well as timely treatment, should be routinely performed in the management and control of TB after HSCT. Our study may help to target high-risk patients for the early-diagnosis and timely treatment making.

## Supplementary information

Supplementary information
