# Supplementary material for: Allo-HSCT recipients with invasive fungal disease and ongoing immunosuppression have a high risk for developing tuberculosis
Source: Sci Rep. 2019 Dec 31;9:20402. doi: 10.1038/s41598-019-56013-w (PMC6938515; doi:10.1038/s41598-019-56013-w)
Supplement: Supplementary file 1 — Supplementary information [file 41598_2019_56013_MOESM1_ESM.docx]

# Supplementary Table

Table S1. Clinical characteristics of patients with TB.

| **patient NO** | **Age/Sex** | **Underlying disease** | **The year of HSCT** | **Donor type** | **aGVHD** | **cGVHD** | **Therapeutic approach of GVHD** | | **Relapse of hematologic disease,Y/N** | [**clinical manifestations**](http://dict.cnki.net/dict_result.aspx?searchword=%e4%b8%b4%e5%ba%8a%e8%a1%a8%e7%8e%b0&tjType=sentence&style=&t=clinical+manifestations) | **site of infection** | **diagnosis of tuberculosis** | **Chest CTscan** | **T-SPOT** | | **Time of Dx of TB after HSCT,day** | **Category of TB** | **Treatment/Outcome of TB** | **disease development** |
| --- | --- | --- | --- | --- | --- | --- | --- | --- | --- | --- | --- | --- | --- | --- | --- | --- | --- | --- | --- |
|  |  |  |  |  |  |  | **Firstline** | **second line** |  |  |  |  |  | **Pre-transplant** | **After transplantation** |  |  |  |  |
| **1** | **23/M** | **ALL** | **2012** | **Haploidentical-related donor** | **IV** | **None** | **steroid pulse therapy, FK506 based** | **etanercept, Simulect** | **N** | **low fever, lumbar soas abscess** | **spine** | **none** | **none** | **Negative** | **Positive** | **86** | **proven** | **Given/ Successful** | **CR** |
| **2** | **17/M** | **CML** | **2012** | **Unrelated donor** | **II** | **None** | steroid pulse therapy, FK506 based | **etanercept** | **N** | **fever, dry cough, emaciation** | **lung** | **lung puncture biopsy** | **mutiple-tubercle shadow,**  **lymphadenectasis** | **Not done** | **Not done** | **43** | **proven** | **Given/ Successful** | **CR** |
| **3** | **18/M** | **AML** | **2013** | **Matched sibling donors** | **0** | **moderate (liver)** | steroid pulse therapy, CSA based | **None** | **N** | **lymphadenectasis** | [**lymph node**](http://dict.cnki.net/javascript:showjdsw('jd_t','j_')) | **lymph node biopsy** | **none** | **Negative** | **Positive** | **909** | **proven** | **Given/ Successful** | **CR** |
| **4** | **38/M** | **AML** | **2013** | **Haploidentical-related donor** | **0** | **moderate (skin, mouth)** | steroid pulse therapy, FK506 based | **etanercept** | **N** | **fever,Cough, expectoration, hemoptysis.** | **lung** | **positive sputum smear** | **milliary pattern** | **Not done** | **Positive** | **261** | **proven** | **Given/ unsuccessful** | **respiratory failure/death** |
| **5** | **60/M** | **AML/MDS** | **2014** | **Matched sibling donors** | **0** | **Severe (eye, liver, skin)** | steroid pulse therapy, CSA based | **None** | **N** | **fever,Cough** | **lung** | **positive sputum smear** | **pneumonia, lenticular shadow, pleural effusion** | **Negative** | **Positive** | **129** | **proven** | **Given/ unsuccessful** | **respiratory failure/death** |
| **6** | **21/F** | **AML** | **2014** | **Unrelated donor** | **IV** | **None** | steroid pulse therapy, FK506 based | **Etanercept ,**  **Simulect** | **N** | **hyperthermia** | **lung** | **none** | **milliary pattern** | **Negative** | **Negative** | **50** | **probable** | **Given/ unsuccessful** | **respiratory failure/death** |
| **7** | **35/M** | **HAL** | **2014** | **Haploidentical-related donor** | **II** | **moderate (skin,liver)** | steroid pulse therapy, FK506 based | **None** | **N** | **fever,Cough** | **lung** | **lung puncture biopsy,positive sputum smear** | **Proliferative lesions** | **Not done** | **Negative** | **231** | **proven** | **Given/ Successful** | **CR** |
| **8** | **45/F** | **NHL** | **2014** | **Haploidentical-related donor** | **III** | **moderate (skin,liver)** | steroid pulse therapy, CSA based | **None** | **Y** | **Cough, breathlessness** | **lung** | **none** | **pneumonia, lenticular shadow,**  **pleural effusion** | **Negative** | **Positive** | **127** | **probable** | **Given/ Successful** | **relapse/death** |
| **9** | **39/F** | **AML** | **2016** | **Haploidentical-related donor** | **0** | **None** | steroid pulse therapy, FK506 based | **None** | **N** | **fever,Cough, expectoration, perinephric abscess** | lung, perirenal tissue | **none** | **milliary pattern** | **Negative** | **Positive** | **220** | **probable** | **Given/ unsuccessful** | **engraftment failure/ multi-organ failure/death** |
| **10** | **49/F** | **AML** | **2017** | **Matched sibling donors** | **0** | **None** | steroid pulse therapy, CSA based | **None** | **N** | **low fever,cough, emaciation, night sweat** | **lung** | **lung puncture biopsy** | **pneumonia** | **Negative** | **Positive** | **167** | **proven** | **Given/ ongoing** | **CR** |
| **11** | **22/M** | **ALL** | **2017** | **Haploidentical-related donor** | **III** | Severe （skin, mouth, liver） | steroid pulse therapy, CSA based | **etanercept** | **N** | **fever,Cough** | **lung** | **none** | **pneumonia** | **Not done** | **Positive** | **370** | **probable** | **Given/ ongoing** | **CR** |
| **12** | **32/M** | **AML(CR2)** | **2017** | **Haploidentical-related donor** | **0** | **None** | steroid pulse therapy, FK506 based | **None** | **N** | **fever,Cough, chest pain, pericardial effusion** | **lung** | **none** | **milliary pattern** | **Negative** | **Positive** | **286** | **proven** | **Given/ ongoing** | **CR** |
| **13** | **9/F** | **AML** | **2017** | **Unrelated donor** | **IV** | **None** | steroid pulse therapy, FK506 based | **Etanercept , Simulect** | **N** | **fever,Cough, hemoptysis** | **lung** | **positive sputum smear and positive sputum cultures** | **pneumonia, lenticular shadow, pleural effusion** | **Negative** | **Positive** | **272** | **proven** | **Given/ Multi-drug resistance/unsuccessful** | **respiratory failure/death** |
| **14** | **22/M** | **ALL(CR2)** | **2017** | **Haploidentical-related donor** | **IV** | **None** | steroid pulse therapy, FK506 based | **Etanercept, Ruxolitinib** | **N** | **intermittent low fever** | **lung** | **Positive sputum cultures** | **mutiple-tubercle shadow** | **Not done** | **Positive** | **50** | **proven** | **Given/ ongoing** | **CR** |
